# Mental imagery-induced attention modulates pain perception and cortical excitability

**DOI:** 10.1186/s12868-015-0146-6

**Published:** 2015-03-15

**Authors:** Magdalena Sarah Volz, Vanessa Suarez-Contreras, Andrea L Santos Portilla, Felipe Fregni

**Affiliations:** Laboratory of Neuromodulation, Department of Physical Medicine and Rehabilitation, Spaulding Rehabilitation Hospital and Massachusetts General Hospital, Harvard Medical School, 125 Nashua Street #727, Boston, 02114 MA USA; Charité - Universitätsmedizin, Berlin, Germany; Department of Neurology, Beth Israel Deaconess Medical Center, Harvard Medical School, Boston, MA USA

**Keywords:** Pain, Attention, Mental imagery, Pain catastrophizing, Cortical excitability

## Abstract

**Background:**

Mental imagery is a powerful method of altering brain activity and behavioral outcomes, such as performance of cognition and motor skills. Further, attention and distraction can modulate pain-related neuronal networks and the perception of pain. This exploratory study examined the effects of mental imagery-induced attention on pressure pain threshold and cortical plasticity using transcranial magnetic stimulation (TMS). This blinded, randomized, and parallel-design trial comprised 30 healthy right-handed male subjects. Exploratory statistical analyses were performed using ANOVA and t-tests for pain and TMS assessments. Pearson’s correlation was used to analyze the association between changes in pain threshold and cortical excitability.

**Results:**

In the analysis of pain outcomes, there was no significant interaction effect on pain between group versus time. In an exploratory analysis, we only observed a significant effect of group for the targeted left hand (ANOVA with pain threshold as the dependent variable and time and group as independent variables). Although there was only a within-group effect of mental imagery on pain, further analyses showed a significant positive correlation of changes in pain threshold and cortical excitability (motor-evoked potentials via TMS).

**Conclusions:**

Mental imagery has a minor effect on pain modulation in healthy subjects. Its effects appear to differ compared with chronic pain, leading to a small decrease in pain threshold. Assessments of cortical excitability confirmed that these effects are related to the modulation of pain-related cortical circuits. These exploratory findings suggest that neuronal plasticity is influenced by pain and that the mental imagery effects on pain depend on the state of central sensitization.

## Background

Mental imagery is the process of envisioning specific physical or cognitive activities or perceptual experiences with the intention of altering the facilitation of neuronal networks [1]. Mental imagery is a powerful tool in improving the performance of motor skills [2-4], cognitive performance, and memory [5] and is widely used in psychological/psychiatric treatments for such disorders as schizophrenia, social phobia, and post-traumatic stress disorder [1].

**Figure 1:**
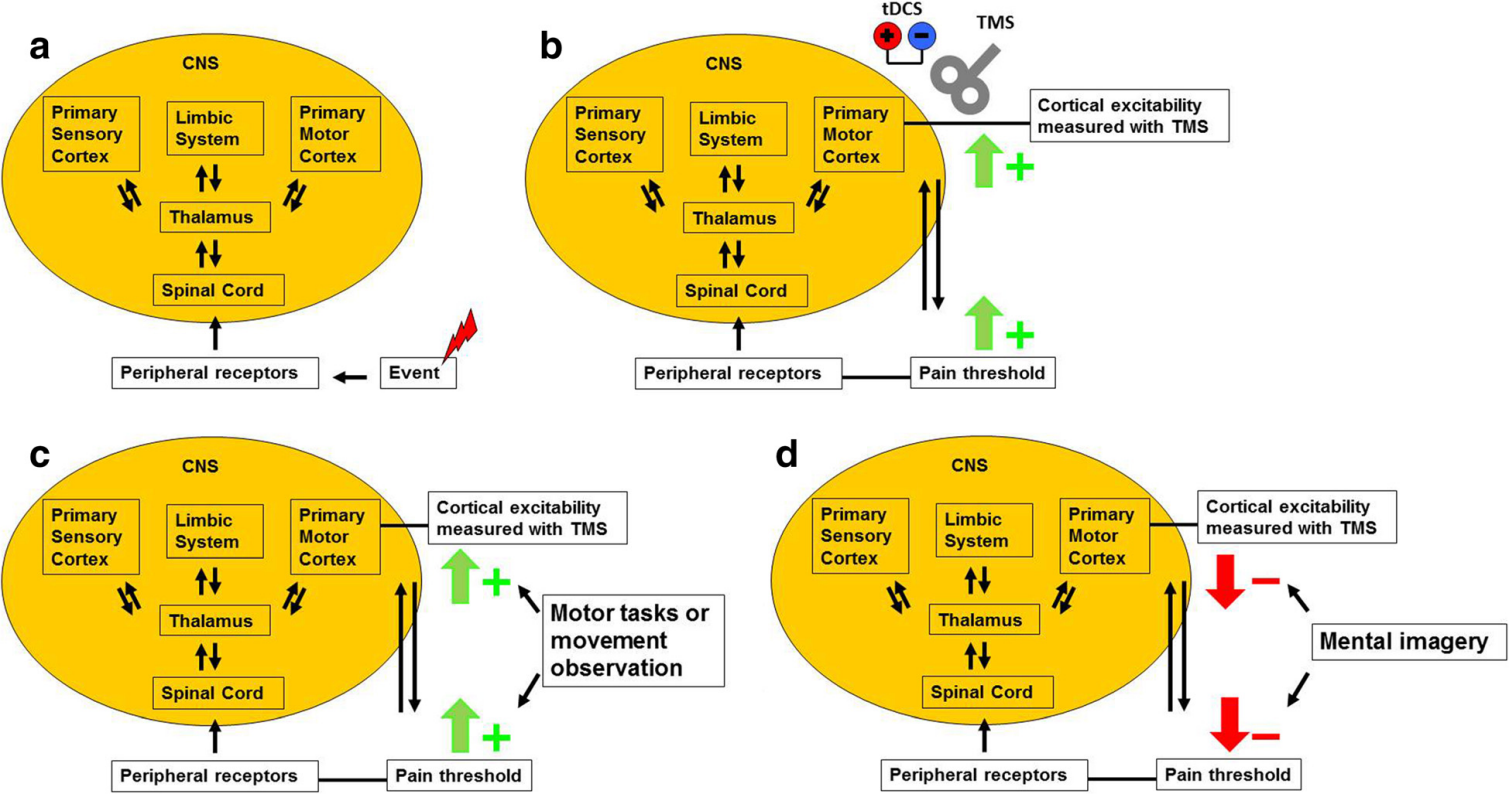
**Relationship between pain perception and motor cortex excitability. a:** Relationship and pathway of pain perception and motor cortex excitability. **b:** Transcranial magnetic stimulation (TMS) and transcranial direct current stimulation (tDCS) can increase motor cortical excitability and pain threshold (= > decrease in pain perception). **c:** Motor tasks and motor observation increase pain threshold and motor excitability. **d:** Mental imagery decreases pain threshold and motor cortical excitability. Pictures modified from Flor [8]; Fregni et al. [9]; and Volz et al. [10].

Mental imagery modulates pain, and certain chronic pain syndromes are altered significantly by mental imagery, such as phantom limb pain [6]. The motor cortex is one neural circuit that can be altered with mental imagery to affect pain sensation (Figure 1a and d). There is increasing evidence of the relationship between the motor cortex and pain modulation (Figure 1a) [7].

Based on data from invasive and noninvasive brain stimulation techniques, such as transcranial magnetic stimulation (TMS) (Figure 1b), there is a bidirectional relationship between pain and motor cortex excitability. Pain perception modifies TMS-indexed cortical excitability in various areas of the brain, including the motor cortex [10-14] (Figure 1a), and modification of motor cortex excitability with repetitive TMS mitigates pain [12,15,16] (Figure 1b). Thus, we hypothesized that mental imagery that is focused toward a specific hand will significantly modify TMS-indexed cortical excitability and pain perception as indexed by quantitative sensory testing (Figure 1d).

Several studies have demonstrated enhanced cortical excitability during mental imagery tasks by measuring motor-evoked potentials (MEPs) using TMS [17-19]. Thus, mental imagery is a proven cognitive tool that changes neuronal plasticity, allowing potential changes in cortical excitability to be correlated with modulations in pain.

In this study, we tested whether mental imagery-induced attention toward a painful stimulus of the hand alters the perception of pain, as measured by pressure pain threshold. We also measured changes in cortical excitability using transcranial magnetic stimulation (TMS) to examine the neurophysiological mechanisms of pain attention. Based on the number of outcomes in this study, we tested this hypothesis in an exploratory manner.

## Results

None of the participating subjects experienced any adverse effects. Because our experimental setting demanded good compliance and attention, we monitored *sleepiness* and *engagement* during the experiment using a questionnaire. One subject was excluded from the experiment, because he fell asleep several times, and experimental adherence was not secured. There were no significant differences between groups in *sleepiness* or *engagement* (*sleepiness*: mental imagery group: 3.71 ± 2.46, control: 3.2 ± 2.78, p = 0.6; *engagement:* mental imagery group: 6.0 ± 2.61, control: 7.6 ± 1.76, p = 0.06; unpaired *t*-test).

Moreover, we studied various age groups (18–40 years and 41–62 years) and found no significant differences (*t*-test: p > 0.05, two-tailed unpaired *t*-test) in pain or TMS outcome. Furthermore we assessed whether there were baseline differences as well as treatment related differences in VAS-anxiety and motor function as indexed by Purdue Pegboard test. All these analyses did not show significant results, confirming that these variables could not explain our results (See Table 1 for statistical details).

**Table 1:** **Statistical analyses of results of VAS-anxiety and motor function as indexed by Purdue pegboard test**

**VAS for anxiety**			
		**Baseline/pre intervention**	**Post intervention**
	Mental imagery group	1.32 ± 1.20	0.96 ± 1.25
	Control group	0.8 ± 0.94	0.43 ± 0.62
	Two-tailed unpaired *t*-test comparing values between groups	P = 0.20	P = 0.13
**Motor function as indexed by the Purdue pegboard test**			
	**Left hand**		
		**Baseline/pre intervention**	**Post intervention**
	Mental imagery group	11.9 ± 2.5	12.24 ± 2.26
	Control group	11.98 ± 2.45	12.02 ± 2.51
	Two-tailed unpaired *t*-test comparing values between groups	P = 0.98	P = 0.79
	**Right hand**		
		**Baseline/pre intervention**	**Post intervention**
	Mental imagery group	12.74 ± 2.49	13.33 ± 2.48
	Control group	12.89 ± 2.44	13.27 ± 2.50
	Two-tailed unpaired *t*-test comparing values between groups	P = 0.87	P = 0.95

Visual analog scale = VAS. Expressed as: mean ± standard deviation.

### Pain threshold

**Figure 2:**
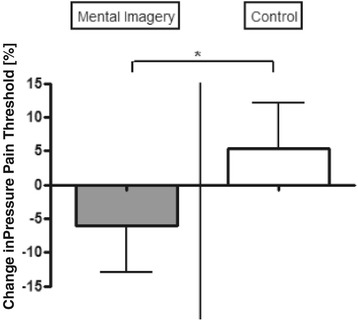
**Pain threshold.** Pain threshold levels for mental imagery and control groups. Ordinate: Changes in pressure pain threshold level as percentage with respect to baseline value (expressed as: [(t2-t1)/t1] ×100). *p < 0.05 as tested with ANOVA (F(1,27) = 7.40, p = 0.0079) with pressure pain threshold as the dependent variable and group (mental imagery vs control) and time (pre- vs post-intervention) as independent variables. Note that the interaction analyses did not reveal significant results.

We analyzed the primary outcome of pain threshold as follows:

i. We initially tested all pain results together. By ANOVA with multiple factors for time, group, and hand, there were no significant interactions (p > 0.05) (Table 2). However, group had a main effect (F _(1,27)_ = 7.40, p = 0.0079), confirming our initial hypothesis that mental imagery-induced attention has a significant effect on the perception of pain, regardless of hand and time (Figure 2).
ii. We then analyzed both hands separately, because only the left hand was targeted in our mental imagination experiment—the right hand served as an intraindividual control condition. We noted a significant result for the left hand for group (ANOVA, F_(1,27)_ = 6.35, p = 0.018), indicating that mental imagery versus controls had disparate effects on pain thresholds. We repeated the same analysis for the right hand (which was not targeted in the experiment) and found no significant results (ANOVA, F_(1,27)_ = 1.56, p = 0.22), confirming that the effects of pain threshold changes in the target hand were induced by the intervention (Table 2).
iii. Pain threshold of the left hand changed in the mental imagery group by-0.63 kg (n = 30; pre: 13.12 kg ± 2.06 kg; post: 12.48 kg ± 2.90 kg) versus +0.24 kg in the control group (n = 30; pre: 14.12 kg ± 4.54 kg; post: 14.36 kg ± 4.18 kg). However, by unpaired *t*-test, this difference was not significant (t-tests: left hand: p = 0.17; right hand: p = 0.59). Note that the baseline thresholds did not differ in either hand in any group (t-tests: left hand: p = 0.70; right hand: p = 0.86).

**Table 2:** **Values of statistical analyses using ANOVA**

**Pain outcome both hands**			**TMS outcome: MEP**		
**Factors**	**Degree of freedom**	**p-Value**	**Factors**	**Degree of freedom**	**p-Value**
Time (pre vs. post) Group (mental imagery vs. control) Hand (right vs. left)	F_(1,28)_ = 7.40	**0.0079**	Time (pre vs. post) Group (mental imagery vs. control)	F_(1,26)_ = 7.93	**0.0091**
Interaction of time and hand	F_(1,27)_ = 1.43	0.2359	Interaction of time and group	F_(1,26)_ = 7.99	0.3753
Interaction of time and group	F_(1,27)_ = 0.47	0.4947			
Time (pre vs. post) Hand (right vs. left)	F_(1,27)_ = 2.87	**0.0939**			
			**TMS outcome: CSP 110%**		
			Time (pre vs. post) Group (mental imagery vs. control)	F_(1,27)_ = 17.40	**0.0003**
			Interaction of time and group	F_(1,27)_ = 0.2	0.6611
**Pain outcome left hand**					
Group(mental imagery vs. control) Time (pre vs. post)	F_(1,27)_ = 6.34	**0.0178**	**TMS outcome: CSP 120%**		
Interaction of time and group	F_(1,27)_ = 0.84	0.3679	Time (pre vs. post) Group (mental imagery vs. control)	F_(1,27)_ = 63.57	**0.0001**
			Interaction of time and group	F_(1,27)_ = 0.3	0.5883
**Pain outcome right hand**			**TMS outcome: CSP 130%**		
Group(mental imagery vs. control) Time (pre vs. post)	F_(1,27)_ = 2.89	0.1001	Time (pre vs. post) Group (mental imagery vs. control)	F_(1,27)_ = 58.35	**0.00001**
Interaction of time and group	F_(1,27)_ = 0.01	0.9248	Interaction of time and group	F_(1,27)_ = 0.74	0.3966
			**TMS outcome: ICF**		
			Time (pre vs. post) Group (mental imagery vs. control)	F_(1,27)_ = 9.86	**0.004**
			Interaction of time and group	F_(1,27)_ = 0.62	0.4363
			**TMS outcome: SICI**		
			Time (pre vs. post) Group (mental imagery vs. control)	F_(1,27)_ = 1.76	0.1948
			Interaction of time and group	F_(1,27)_ = 0.29	0.5919

Cortical silent periods = CSP; Motor-evoked potentials = MEP; Short intracortical inhibition = SICI; Intracortical facilitation = ICF.

### Transcranial magnetic stimulation

Because we targeted the left hand in the experiments, TMS was assessed only over the right nondominant hemisphere. All TMS analysis results are listed in Table 2.

We first tested whether cortical excitability changed significantly with of group and time as factors by ANOVA (hand was not a factor, because only one hemisphere was examined). By ANOVA, there were significant results for both groups (MEP amplitude: F_(1,26)_ = 7.93, p = 0.0091).

**Figure 3:**
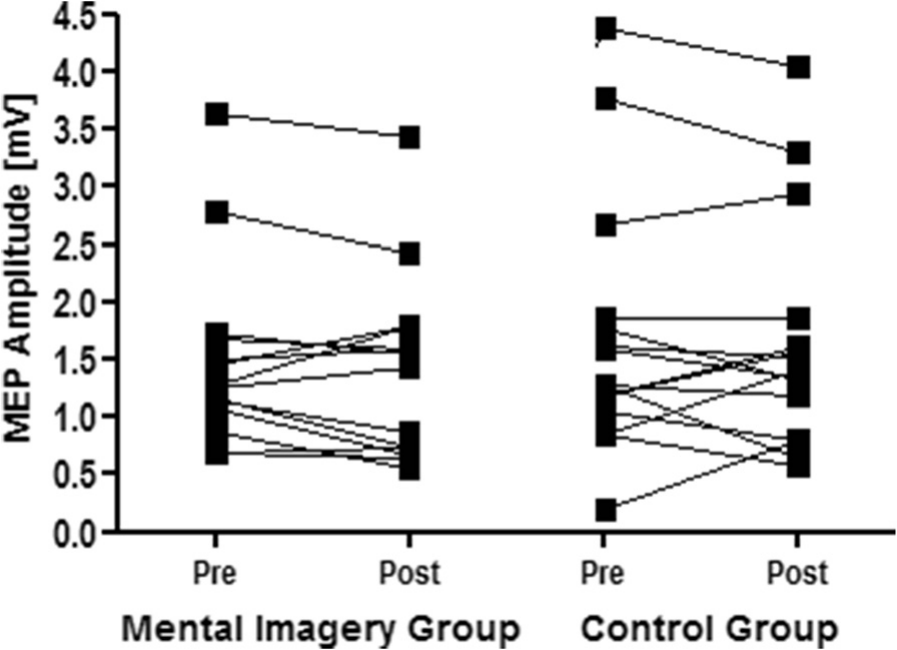
**Motor-evoked potentials.** MEP amplitudes in mV of both groups pre- and postintervention.

Notably, baseline values did not differ between groups with regard to MEP amplitude [p = 0.597 (pre intervention values: mental imagery group: 1.493 mV ± 0.809 mV; controls: 1.700 mV ± 1.117 mV; post intervention: mental imagery group: 1.414 mV ± 0.813 mV; controls: 1.663 mV ± 1.009 mV)] and MEP integral [p = 0.816 (pre intervention: mental imagery group: 0.0214 mV*s ± 0.0141 mV*s; controls: 0.0224 mV*s ±0.0169 mV*s; post intervention: mental imagery group: 0.0193 mV*s ± 0.0127 mV*s; controls: 0.0218 mV*s ±0.0155 mV*s;)], indicating that the effects were not due to a baseline difference between groups. Overall, MEP decreased over time (mental imagery group: from 1.493 mV to 1.414 mV). Expressed as percentages; MEP in the mental imagery group decreased by 5.33% versus 2.2% in the control group. Individual changes in MEP are shown in Figure 3.

**Figure 4:**
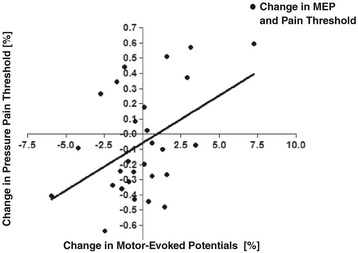
**Correlation of changes in pain thresholds and MEP amplitude.** Correlation between pain threshold of the left hand and MEP amplitudes. Ordinate: Change in pressure pain threshold [in %]. Abscissa: Change of MEP amplitude [in %]; (r = 0.46; p = 0.015). Changes in pressure pain threshold were calculated as follows: t_2_ (postintervention value) – t_1_ (preintervention value). Changes in MEP amplitude were calculated as follows: t_2_ (postintervention amplitude) – t_1_ (preintervention amplitude).

We then determined whether the changes in pain threshold level were associated with modulations in cortical excitability by correlation analysis. Shifts in pain threshold and MEP amplitude correlated significantly (Pearson’s correlation: r = 0.46; p = 0.015), suggesting that a decline in pain threshold (ie, greater sensitivity to pain perception) decreases cortical excitability (Figure 4).

The mean and standard deviation of MEP amplitude and integral before and after the intervention of both groups are listed in Table 3. MEP amplitudes in mV pre- and post-intervention of both groups are shown as graphs in Figure 3.

**Table 3:** **Results of motor-evoked potentials**

**Mental imagery group**					**Control group**				
**Time**	**Pre**		**Post**		**Time**	**Pre**		**Post**	
**Subject**	**Amplitude**	**Integral**	**Amplitude**	**Integral**	**Subject**	**Amplitude**	**Integral**	**Amplitude**	**Integral**
**Number**	**(mV)**	(mV*s)	**(mV)**	(mV*s)	**Number**	**(mV)**	(mV*s)	**(mV)**	(mV*s)
1	0.68	0.01	0.73	0.01	3	2.68	0.04	2.94	0.04
2	1.25	0.01	1.43	0.01	6	1.85	0.03	1.87	0.03
4	MD	MD	MD	MD	7	0.85	0.01	1.42	0.01
5	1.28	0.01	1.79	0.03	9	1.28	0.01	0.64	0.01
8	0.86	0.01	0.55	0.01	14	1.19	0.01	1.56	0.02
10	1.46	0.02	1.80	0.03	15	1.58	0.02	1.34	0.02
11	1.51	0.02	1.60	0.02	16	1.62	0.02	1.53	0.02
12	1.67	0.03	1.60	0.02	17	1.18	0.02	1.62	0.03
13	1.16	0.02	0.73	0.00	19	4.38	0.06	4.04	0.05
18	1.07	0.02	0.66	0.01	21	0.84	0.01	0.57	0.01
20	2.78	0.04	2.42	0.03	22	1.76	0.02	1.31	0.01
24	1.72	0.02	1.54	0.02	23	3.77	0.06	3.29	0.05
25	0.70	0.01	0.64	0.01	27	0.20	0.00	0.80	0.01
26	3.63	0.06	3.43	0.05	28	1.28	0.01	1.18	0.01
29	1.14	0.02	0.86	0.01	30	1.06	0.01	0.81	0.01
**Mean**	1.49	0.02	1.41	0.02	**Mean**	1.70	0.02	1.66	0.02
**SD**	0.81	0.01	0.81	0.01	**SD**	1.12	0.02	1.01	0.02

Baseline values did not differ between groups: MEP amplitudes: *t*-test: p = 0.597 (mental imagery group: mean: 1.493 mV ± 0.809 mV; controls: mean: 1.700 mV ± 1.117 mV); MEP integral: *t*-test: p = 0.816 (mental imagery group: mean: 0.0214 mV*s ± 0.0141 mV*s; controls: mean: 0.0193 mV*s ±0.0127 mV*s).

Mean and standard deviation of MEP amplitudes and integral before and after the intervention in both groups (MD = missing data).

We analyzed whether the effects were due to the experimental group. By ANOVA with of group and time as factors for all cortical silent periods (CSP) with intensities of 110% (mental imagery group: pre intervention: 0.069 s ± 0.032 s; post intervention: 0.07 s ± 0.035 s; control group: pre intervention: 0.065 s ± 0.014 s; post intervention: 0.068 s ± 0.019 s), 120% (mental imagery group: pre intervention: 0.085 s ± 0.039 s; post intervention: 0.087 s ± 0.041 s; control group: pre intervention: 0.082 s ± 0.022 s; post intervention: 0.087 s ± 0.024 s), and 130% (mental imagery group: pre intervention: 0.095 s ± 0.043 s; post intervention: 0.097 s ± 0.044 s; control group: pre intervention: 0.099 s ± 0.028 s: post intervention: 0.106 s ± 0.029 s), there were significant results for group, confirming our findings from the pain analysis (ANOVA for CSP 110%: F_(1,27)_ = 17.40, p = 0.0003; ANOVA for CSP 120%: F_(1,29)_ = 63.57, p = 0.0001; ANOVA for CSP 130% F_(1,27)_ = 58.35, p = 0.00001). This result indicates that the groups differed significantly in changes in TMS measures. By ANOVA of ICF, we noted significant results for the experimental group (F_(1,27)_ = 9.86, p = 0.0040), demonstrating that the cortical excitability in the mental imagery group changed disparately than in the control group.

SICI was unchanged in both groups using t-tests (p > 0.05 for all analyses) as well as interaction analyses (ANOVA).

We also performed linear regression analyses to test for confounders, but none revealed any significant results (p > 0.05). Thus, potential confounders, such as age, race, education level, state of engagement and sleepiness, anxiety level, and motor function ability (ie, Purdue pegboard test), did not influence the results. No subject had a score that was higher than 6 (out of 63, mean score: 0.6 ± 1.6), reflecting the absence of depressive symptoms (per [20]: a score between 11–17/63 indicates mild depressive symptoms, and a score over 18/63 is defined as clinically relevant).

## Discussion

In this exploratory study, we did not observe any significant effect of mental imagery on pain in our primary analysis. However, we noted a small effect in the exploratory within-group analyses. The targeted left hand experienced a decrease in pressure pain threshold, indicating a rise in pain perception. In contrast, pressure pain threshold rose in the controls, although, this within-group effect was modest. Further analyses of TMS assessments revealed a significant positive correlation of changes in pain and alterations in cortical excitability, suggesting that a decline in pain threshold decreases cortical excitability.

Our results can be interpreted as unexpected, because the literature claims that MEPs increase significantly during voluntary or imagined movement of the finger [21]. Also, studies on phantom limb pain suggest that the use of mental imagery is an effective method of reducing pain [22-25]. Nevertheless, our findings demonstrate the opposite.

One explanation is that we included healthy subjects with an experimental pain model—not patients who were suffering from actual pain. Because chronic pain patients have a deficient pain matrix and altered pain-related neural networks, such methods as mental imagery might induce differential effects in patients versus healthy subjects [8,16,25,26]. In previous studies, we found that motor tasks, sensory stimuli, and movement observation can change pressure pain threshold levels and cortical excitability [10,27,28] (Figure 1c), in which active tasks for one hand can ameliorate the perception of pain in the targeted hand. In contrast, we also found that the untargeted hand experienced a decrease in pain threshold, indicating greater perception of pain [10,27].

In the current exploratory study, we generated data that suggest that the opposite effects are occurring, because attention and expecting pain indicated enhanced perception of pain. Thus, our findings implicated a close, reciprocal relationship of these two emotional and alertness states. Consistent with our modest effects on pain, these effects have been confirmed by several studies [29-34].

In a separate study, we demonstrated that changes in pain correlate significantly with modulations in TMS assessments, as in our present report [10,35]. In that study, we tested movement observation by showing a video, which was intended to be a distraction to a painful stimulus [10] (Figure 1c). Pain threshold levels increased, reflecting a decline in the perception of pain [10]. This finding is consistent with our current results, which yielded the opposite effects in both outcomes. Subjects paid attention to the hand and focused on the painful stimuli, which effected a moderate decrease in pressure pain levels, thus indicating pain sensitization. Further, previous evidence has shown that cortical excitability and pain significantly correlate in chronic pain patients [16,36-38], supporting our exploratory findings.

The modest within-group effects seen in this article suggest that complex processes, such as distraction and concentration, and influences of attentional processes have an impact on pain perception [32,39,40]. For instance, paying attention to pain, focusing on a painful body part, and rumination of painful stimuli are components of pain catastrophizing [41]. In our experiment, we mimicked attention to a specific body part and placed the focus on painful stimuli on the same area. Thus, we might be able to mimic one part of the complex mechanism of pain catastrophizing and attention-modified pain perception in healthy subjects.

Moreover, cortical excitability is significantly associated with pain coping strategies, such as pain catastrophizing, supporting our findings that cortical excitability results correlate significantly with pain outcome [35]. A subsequent study with a larger sample size should be performed to determine the predictors of pain catastrophizing and test interventions to prevent attention, focusing, and rumination on pain. The mechanism of pain augmentation must be determined to develop novel targeted therapies for chronic pain conditions.

There are some limitations in our study. First, we assessed TMS on one hemisphere, because only the left hand was the target in our experimental setting—cortical excitability changes were not measured in the other hemisphere. However, because there was no significant effect of pain threshold in the non-targeted right hand, we assumed that there was no such effect on the non-targeted left hemisphere. Further, studies with larger sample sizes that include chronic pain patients are necessary to confirm the results and to show the differences in pain processing between healthy subjects and chronic pain patients.

Also, the age difference between subjects could be a limitation. Although there no significant differences between younger and older subjects, we cannot exclude that age has an influence on our results. In addition, with regard to limitations due to the statistical results: we did not find any significant interactions—only a modest within-group effect on pain outcome was noted.

## Conclusions

Mental imagery-induced attention and focusing on a painful stimulus of a specific body part might enhance the perception of pain. Our findings highlight the effects and influence of attentional processes on pain perception, which might be components of the mechanisms of pain catastrophizing and chronification, because both phenomena include recurring attention on pain and a painful body part. Further, cortical plasticity changes in the same direction as those in pain perception. These exploratory findings suggest that neuronal plasticity is governed by pain and that pain-related neural networks are altered by the attention state.

## Methods

### Experimental design

**Figure 5:**
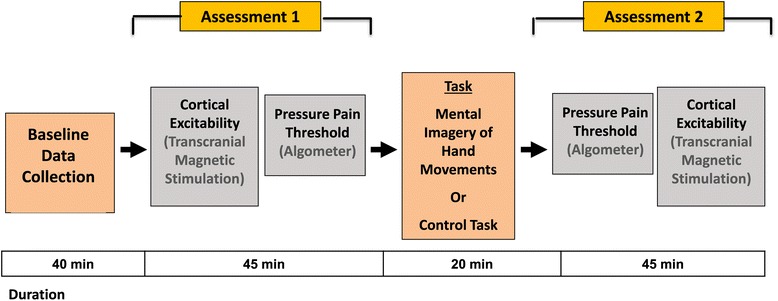
**Study design.** Study design and duration of experiment.

This study was a blinded, randomized, controlled, parallel-design trial. Thirty healthy right-handed male subjects were enrolled. The participants were randomized into 1 of 2 groups (15 volunteers in both study arms; in total, 30 participants). Both groups underwent the same procedures, including determination of pressure pain threshold and measurements of cortical excitability with transcranial magnetic stimulation before and after the intervention (Figure 5). The intervention was mental imagery of hand movements or a control task (see below).

Other assessment scales were administered to control for sleepiness and engagement during the experiment, in addition to the visual analog scale (VAS) for anxiety and the Purdue pegboard test. The VAS for anxiety [42] is a 10-point rating system (with 0 indicating no anxiety and 10 indicating the worst possible anxiety). The same system was used for the sleepiness and engagement questionnaire (with 0 indicating no sleepiness or engagement and 10 indicating the greatest sleepiness or engagement). The Purdue pegboard test assessed motor function throughout the experiment [43]. The questionnaire for sleepiness and engagement was given after the intervention period, pain threshold measures, and TMS assessment.

This study was approved by the institutional review board of Spaulding Rehabilitation Hospital (Harvard Medical School, Boston, USA) and was conducted per the ethical principles of the World Medical Association/Declaration of Helsinki. The ClinicalTrials.gov identifier is NCT01404039.

### Intervention: mental imagery group

Subjects (mean age: 40 years ±12.59 years, range: 19–62 years) were asked to practice mental imagery of motor practice, consisting of sequential and repetitive finger movements of the left hand, for 10 minutes [1,3]. The subjects were seated in a chair and asked to keep their arm and hand muscles fully relaxed, which was first controlled visually by 1 of the experimenters and also controlled by surface electromyographic activity (EMG) recordings.

Participants were instructed to pay attention to the left hand. Further, they were asked to focus on the painful stimulus during the pressure pain threshold measurements, which were performed immediately before and after the period of mental imagery. Subjects were instructed to imagine repetitive movements of the left index finger to the left thumb for 5 minutes (thumb to second finger). Subjects were then asked to consecutively imagine sequential movements of the remaining fingers to the left thumb (thumb to third, fourth, and fifth fingers) for 5 minutes. They were told to concentrate and focus on the left hand. In all subjects, surface (EMG) was recorded simultaneously from the flexor digitorum superficialis (FDS) and opponens pollicis (OP) bilaterally.

### Control group

The control for mental imagery consisted of performing a simple mental calculation, such as adding or subtracting a 1-digit number from a starting number (eg, 1 + 1 = 2; 2 + 1 = 3; 3 + 1 = 4, etc.). In all subjects (mean age: 36.8 years ±14.37 years, range: 20-60 years), EMG activity was recorded simultaneously from the FDS and OP bilaterally. EMG activity was recorded simultaneously from the flexor digitorum superficialis (FDS) and opponens pollicis (OP) bilaterally.

### Subjects

Thirty healthy right-handed male subjects (mean age: 38.1 years ±13.24 years, range: 18–62 years) were recruited through postings in public places and the internet. Participants who fulfilled the following criteria were eligible to participate: (1) male; (2) aged between 18 and 65 years; (3) right-handed, indexed per the Edinburgh Handedness Inventory [44]; (4) no neurological or psychiatric disorders, as assessed by Beck Depression Inventory [20] (mean score: 0.6 ± 1.6); (5) no use of central nervous system medications; (6) no contraindications to TMS [45]; (7) no rheumatologic disease; and (8) no history of alcohol or substance abuse within the last 6 months.

All 30 subjects provided written informed consent. To create a homogeneous study population, we enrolled only right-handed male participants, because female hormones influence cortical excitability and the dominance of the hemisphere [46,47].

### Pain assessment: pressure pain threshold

Pressure pain thresholds were determined with a Commander algometer (JTech Medical Industries, Salt Lake City, USA). The algometer has a 1-cm^2^ rubber probe, which was pressed against the hand (the thenar area of each hand). The applied velocity was 1 kg/cm^2^s. Subjects reported when the pressure stimulus became painful [48]. Because testing pressure pain threshold is operator-dependent, only 1 experienced researcher measured pain to avoid interrater variability and to ensure the same velocity of the increase in pressure. The investigator was blinded to the intervention and unable to view the pressure intensities.

Three repetitions were measured, the thresholds for which were averaged. The area in which pressure was applied minimally differed for each repetition to avoid habituation. One measure took approximately 1 minute per test, totaling roughly 6 minutes. Pressure pain thresholds were determined for both hands. Pain assessments were conducted immediately before and after the period of mental imagery to avoid disrupting the mental imagery process and the subject’s concentration.

### Cortical excitability: transcranial magnetic stimulation (TMS)

TMS was assessed using a Bistim2 stimulator and a figure- eight coil (Magstim Company LTDA, UK). Ag/AgCl electrodes (ADinstruments, Colorado Springs, CO, USA) were placed over the first dorsal interosseus muscle (FDI), and a ground electrode was placed over the subject’s forearm. EMG recordings were processed using Powerlab 4/30 (ADinstruments, Colorado Springs, CO, USA) with a band pass filter of 20–2000 kHz. Offline analyses were performed on a private computer using LabChart (ADinstruments, Colorado Springs, CO, USA). First, head measures were taken to identify the approximate spot of the motor cortex (using the vertex as the reference). Then, the TMS coil was held tangentially over the motor cortex at an angle of 45° with respect to the sagittal line of the head. The hotspot was determined by carefully eliciting the most stable and highest MEP amplitudes over the FDI. The best location was marked with a pen on a swim cap, which was worn by each of the subjects.

We defined the following TMS parameters for the assessments. Cortical silent periods (CSPs) are a measure of intracortical inhibition, changes in which are related to GABA activity [49]. In chronic pain, the CSP declines [50]. Our hypothesis was that mental imagery would increase the CSP. Further, motor-evoked potentials (MEPs) are a direct measure of corticospinal excitability [49] that rise in association with alleviation of pain [51]. We hypothesized that mental imagery would increase MEPs. Short intracortical inhibition (SICI) is believed to be controlled by presynaptic GABA_B_ [49]. We hypothesized that SICI would be enhanced with mental imagery. Intracortical facilitation (ICF) is linked to NMDA receptor activation [49], and we hypothesized that ICF decreases during mental imagery, because it is reduced with pain treatment.

TMS was evaluated on the right hemisphere and the contralateral, left FDI, which reflected the nondominant hemisphere in all subjects. Resting motor threshold (MT) was determined by eliciting 3 of 5 MEPs with a minimal peak-to-peak amplitude of 100 μV [27,52,53]. MEPs were excited with 130% of the individual MT. CSPs were measured at intensities of 110%, 120%, and 130% of the individual MT. Subjects were instructed to perform isometric voluntary contraction during CSP recordings with 15% of maximum contraction force, controlled by a mechanical pinch gauge (Baseline® Evaluation Instruments, Chattanooga, TN, USA) [27,53,54].

TMS measurements included SICI with an interstimulus interval (ISI) of 3 ms and ICF with an ISI of 10 ms [55]. For paired-pulse measurements, the first stimulus was set to 70% of the individual MT, and the second stimulus was set to the individual MEP intensity. Fifteen recordings of each TMS assessment protocol were randomly elicited. Offline analyses included measures of peak-to-peak amplitude, the area-under-the-curve of all MEPs, and the relative duration of CSPs (time from last MEP until normal muscle activity was re-achieved).

### Further assessments

The Beck Depression Inventory (BDI) is a 21-item test that is presented in multiple-choice format that measures the presence and degree of depression in adults [20].

The Purdue pegboard test measures finger dexterity and monitors motor skills by assessing changes over time through the speed of performance [56,57]. The Purdue pegboard test also assesses motor function [43]. The subject is seated comfortably at a normal-height table. A pegboard is placed in front of the person with a row of cups at far end. The cup on the pegboard contains 25 pins that subjects must place in the correct order (starting with the top hole) as fast as possible. Only one pin at a time can be picked up. If a pin is dropped during the test, the subjects should continue picking up another pin. The entire procedure takes 30 seconds. Each participant repeated the task for 3 times, and median was calculated [58].

### Statistical analyses

Data are presented as mean ± standard.

Analyses were performed using STATA (version 11.0, College Station, Texas, US) and GraphPad Prism (version 4.00 for Windows, GraphPad Software, La Jolla, CA, USA).

Mixed ANOVA models with hand, time, and group as factors were used to analyze changes in pain outcome for the effects of hand (left hand, which was the target of mental imagery, and right hand, which was not the target), group (mental imagery versus control group), and time (before and after intervention). TMS data were analyzed with a mixed ANOVA model using measures of cortical excitability (MEP, CSP, SICI, ICF) to test for time (before and after intervention) and group (mental imagery group versus control group) as factors.

Pearson’s correlations were conducted to examine the relationship between changes in motor cortical excitability via TMS measurements and pain outcome, as assessed by pressure pain threshold.

To identify potential confounding variables and to detect any association with dependent (pressure pain threshold) and independent variables (such as age, race, education level, state of engagement and sleepiness, anxiety level, motor function ability—ie, Purdue pegboard test), we performed multiple regression analyses. In addition, two-tailed unpaired t-tests were used to control for differences in baseline characteristics and assessment scores between groups.

Significance was considered at a two-sided level of p < 0.05. We did not correct for the significance threshold in the multiple comparisons, given the exploratory nature of this study and the number of outcomes. For the TMS measurements, based on the number of tests, it is likely that at least 1 of the significant results is due to chance.
